# Asymmetric Transmission in a Mie-Based Dielectric Metamaterial with Fano Resonance

**DOI:** 10.3390/ma12071003

**Published:** 2019-03-27

**Authors:** Xiaobo Wang, Haohua Li, Ji Zhou

**Affiliations:** State Key Laboratory of New Ceramics and Fine Processing, School of Materials Science and Engineering, Tsinghua University, Beijing 100084, China; wangxb14@mails.tsinghua.edu.cn (X.W.); lhh14@mails.tsinghua.edu.cn (H.L.)

**Keywords:** Mie resonance, dielectrics, metamaterial, Fano, asymmetric transmission

## Abstract

Chiral metamaterials with asymmetric transmission can be applied as polarization-controlled devices. Here, a Mie-based dielectric metamaterial with a spacer exhibiting asymmetric transmission of linearly polarized waves at microwave frequencies was designed and demonstrated numerically. The unidirectional characteristic is attributed to the chirality of the metamolecule and the mutual excitation of the Mie resonances. Field distributions are simulated to investigate the underlying physical mechanism. Fano-type resonances emerge near the Mie resonances of the constituents and come from the destructive interference inside the structure. The near-field coupling further contributes to the asymmetric transmission. The influences of the lattice constant and the spacer thickness on the asymmetric characteristics were also analyzed by parameter sweeps. The proposed Mie-based metamaterial is of a simple structure, and it has the potential for applications in dielectric metadevices, such as high-performance polarization rotators.

## 1. Introduction

Optical diodes play a significant role in the application of optoelectronics and optical computing [1,2,3]. Similar to the diodes in electronic components, they show different optical transmissions in two opposite directions. Traditional diode-like optical or electromagnetic devices need to break the time reversal and spatial inversion symmetries simultaneously [4], which requires materials with specific optical properties. Recently, chiral metamaterials, which are able to support asymmetric transmission (AT) for linearly or circularly polarized electromagnetic waves [5,6,7], provide a reciprocal route to mimic the unidirectional transmission feature of diodes. The metamaterial methods allow a free design of the metamolecules with extraordinary physical properties, such as negative refraction [8], reversed Cherenkov radiation [9], and enhanced nonlinearity [10]. Generally, the intriguing electromagnetic characteristics are attributed to the special-designed metallic or dielectric resonators. Metamaterials based on dielectric resonators are believed to show lower heat dissipation in higher frequencies [11,12] when compared with metallic structures. Additionally, Mie resonances are widely used as constructing modes in dielectric-based metamaterials. They can sustain dipoles, quadrupoles, or more complicated patterns [13]. Hence, Mie-based dielectric resonators can give much more freedom in the designing of metamaterials with simple structures.

Fano resonances, which are distinguished by their asymmetric resonance spectra [14,15], have been realized in various metamaterials via a symmetry-breaking method [16,17]. They are commonly interpreted as both constructive and destructive interferences in the near field, producing a sharp spectrum with a high quality factor and a localized field [18,19]. The unique coupling effect and the close connection with symmetry endow applications in switching [20,21] and sensing [22]. Furthermore, utilizing Fano resonances to design metamaterials that exhibit or enhance asymmetric transmission contrast [1,23] can be a feasible strategy.

Currently, most metamaterials with the AT effect are based on rather complicated metallic structures [24,25,26,27,28,29]. Some progress has been made using simple dielectric-based structures [30,31,32]. Jiang et al. [33] demonstrated tunable circular asymmetric transmission in a Mie-type dielectric metasurface integrated with graphene sheets. However, many dielectric chiral structures are still too complex [7,34]. Herein, illuminated by a photonic crystal [34], we have numerically demonstrated a bilayer Mie-based dielectric metamaterial working in the X-band, which exhibits a dual-band AT effect of linearly polarized waves. The chiral metamolecule consists of two identical Mie resonators with high permittivity, which are of a simple structure. The Fano interplay between the constituents was studied. Furthermore, the connection between the AT phenomenon and the Mie resonances was investigated.

## 2. Materials and Methods

For a reciprocal system, considering a linearly polarized TEM (transverse electro-magnetic) wave propagating along the +*z* direction, a Jones Matrix is used to define the relation between the incident and the transmitted electric fields based on a previous study [27,35]:

(1) $$J_{+}=\left(\begin{array}{cc} T_{xx} & T_{xy}\\ T_{yx} & T_{yy} \end{array}\right),$$

where T*_xx_*, T*_xy_*, T*_yx_*, and T*_yy_* are the four elements of the Jones matrix. If the wave propagates in the −*z* direction, the matrix becomes:

(2) $$J_{-}=\left(\begin{array}{cc} T_{xx} & -T_{yx}\\ -T_{xy} & T_{yy} \end{array}\right),$$

Additionally, the AT effect happens when satisfying the condition of:

(3) $$\left|T_{yx}\right|=\left|T_{xy}\right|,$$

It requires the symmetry in the *z* direction to be broken, and chiral structures are commonly utilized to achieve this goal [26,28,36], which is the basis of our design. It should be stressed that the AT phenomenon here are reciprocal in physics.

**Figure 1:**
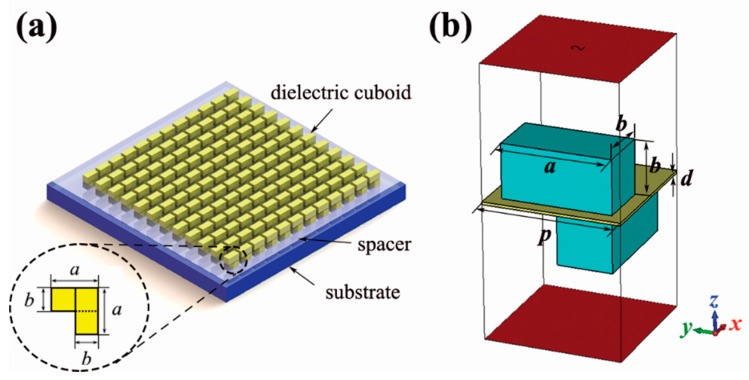
(**a**) Schematic diagram of the proposed chiral metamaterial. The inset illustrates the relative position of two orthogonal cuboids in the metamolecule. (**b**) Simulation settings of the unit cell. The geometrical parameters are: *a* = 4 mm, *b* = 2 mm, *p* = 5 mm, and *d* = 0.1 mm. The receiving and the transmitting ports are in red color.

Figure 1a is the schematic diagram of the proposed metamaterial, and Figure 1b depicts the geometry of the unit cell for simulation. The complex permittivity of the dielectric resonators is set as 155 + 0.3i, a typical value for the CaTiO_3_-1 wt.% ZrO_2_ ceramic in the X-band [37]. The chiral metamolecule is comprised of two identical ceramic cuboids. The cuboids are separated by a low-index FR-4 spacer (*ε* = 4.2), which is 0.1 mm thick. The dimensions of the cuboids are 4 mm, 2 mm, and 2 mm, respectively. The lattice constant of the metamaterial is *p* = 5 mm, which is less than 1/6 of the operating wavelength. The incident electric field polarizes in the *x* direction. Periodic boundary conditions are applied on the *x* and the *y* boundaries to form an array. It should be mentioned that the substrate illustrated in Figure 1a was not considered for the simplicity of simulation.

The simulations were carried out using a commercial finite-element software package CST Microwave Studio 2013 (Computer Simulation Technology). We began by emulating the spectra of S-parameters when the incident wave propagated in the +*z* and the −*z* directions. To calculate the full transmissions, four S-parameters are required according to:

(4) $$\{\begin{array}{c} T_{f}={\left|S_{xx}\right|}^{2}+{\left|S_{xy}\right|}^{2}\\ T_{b}={\left|S_{yy}\right|}^{2}+{\left|S_{yx}\right|}^{2} \end{array},$$

where S*_ij_* (simplified from S_*i*2, *j*1_) are the S-parameters (*i*, *j* = *x*, *y*), representing the ratio of the transmitted electric field’s *i* component and the *j*-polarized incident electric field; *T_f_* and *T_b_* are the forward (+*z*) and the backward (−*z*) transmittance. S*_xx_*, S*_xy_*, S*_yx_*, and S*_yy_* correspond to the *T_xx_*, *T_xy_*, *T_yx_*, and *T_yy_* in the Jones Matrix, respectively. In our case, S*_xx_* is equal to S*_yy_*. Then, the AT parameter to characterize the performance was calculated by [26]:

(5) $$\Delta_{x}={\left|S_{yx}\right|}^{2}-{\left|S_{xy}\right|}^{2}=-\Delta_{y}$$

where Δ*_x_* and Δ*_y_* are AT parameters for the *x*-polarized and the *y*-polarized wave. Next, the magnetic field distributions at the peaks of S*_xy_* and S*_yx_* were simulated to clarify the physical mechanism. 

**Figure 2:**
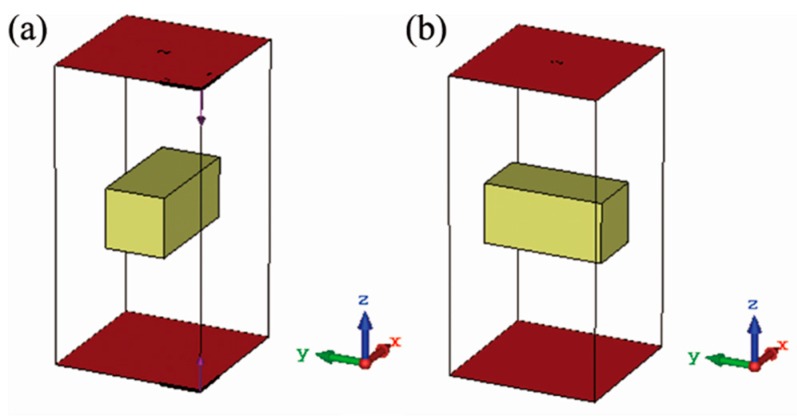
Simulation settings of the two single dielectric cuboids. (**a**) Cuboid 1. (**b**) Cuboid 2. The red facets are the receiving and the transmitting ports.

Furthermore, the transmission responses of the two single cuboids in the metamolecule were also calculated to determine their Mie resonances. The simulations were performed using the configurations illustrated in Figure 2a,b. PEC (perfect electric conductor) and PMC (perfect magnetic conductor) boundary conditions were applied on the *x* and the *y* boundaries, respectively. The electromagnetic wave propagated along the *z* direction, and the polarization of its electric field was in the *x* direction. The transmission was characterized by S_21_. S_21_ should be roughly viewed as S*_xx_* because the *y* component of the transmitted electric field is small enough in this situation.

Finally, the parameter sweeps of the lattice constant and the dielectric spacer’s thickness were performed to investigate the coupling effect and acquire the optimal configurations.

## 3. Results and Discussion

**Figure 3:**
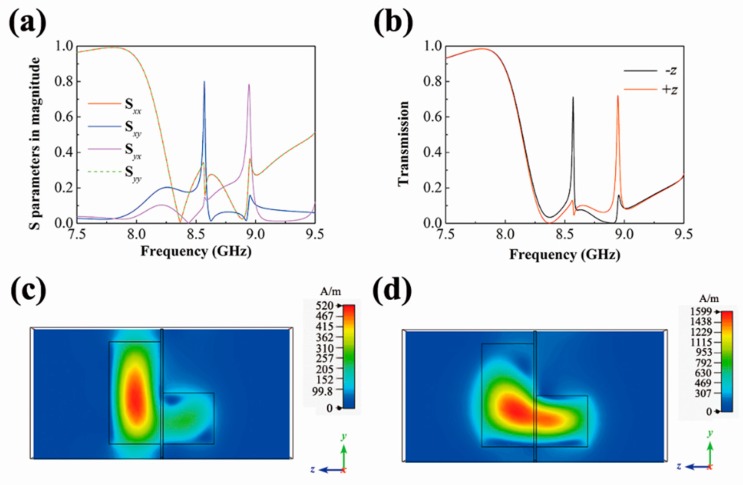
(**a**) Simulated spectra of the four S-parameters. (**b**) Calculated transmissions in the -*z* and the +*z* directions. Simulated magnetic field |*H*| at 8.571 GHz in the *y*-*z* plane under a (**c**) *x*-polarized and (**d**) *y*-polarized incident wave.

Figure 3a illustrates the simulated S-parameters’ (S*_xx_*, S*_xy_*, S*_yx_*, S*_yy_*) spectra of the chiral metamaterial. Two asymmetric Fano-type resonances are seen at 8.571 GHz in the S*_xy_* spectra and at 8.944 GHz in the S*_yx_* spectra. The curve of S*_xx_* is nearly the same as that of S*_yy_*, while S*_xy_* is unequal to S*_yx_* in the vicinity of the two Fano-like resonance peaks. Besides, the peak of the S*_xy_* curve suggests the structure can convert the *y*-polarized incident wave into a mainly *x*-polarized transmitted wave. Additionally, the rotation of polarization is different at the peak of S*_yx_*. Similarities can be found in previous research based on a complex metallic structure [38]. These results make the proposed metamaterial suitable for rotators after proper modifications. 

The calculated transmission spectra in the +*z* and the −*z* directions are presented in Figure 3b. The transmission peak in the curve of the −*z* direction is at 8.569 GHz and the peak value is 0.713, while the transmission in the +*z* direction is 0.102 at the same frequency. Additionally, the peak value in the +*z* direction is 0.722 at 8.946 GHz compared with a transmission of 0.125 in the −*z* direction. The results indicate a dual-band AT effect. One can assume the two Fano-type resonances may contribute to the AT phenomenon since the peaks of the calculated transmission are quite near the asymmetric Fano peaks of S*_xy_* and S*_yx_*. In addition, the transmission peak at 8.569 GHz also gives a sharp Fano line shape in Figure 3b. Thus, this Fano-like mode accompanied by the S*_xy_* peak was further analyzed.

**Figure 4:**
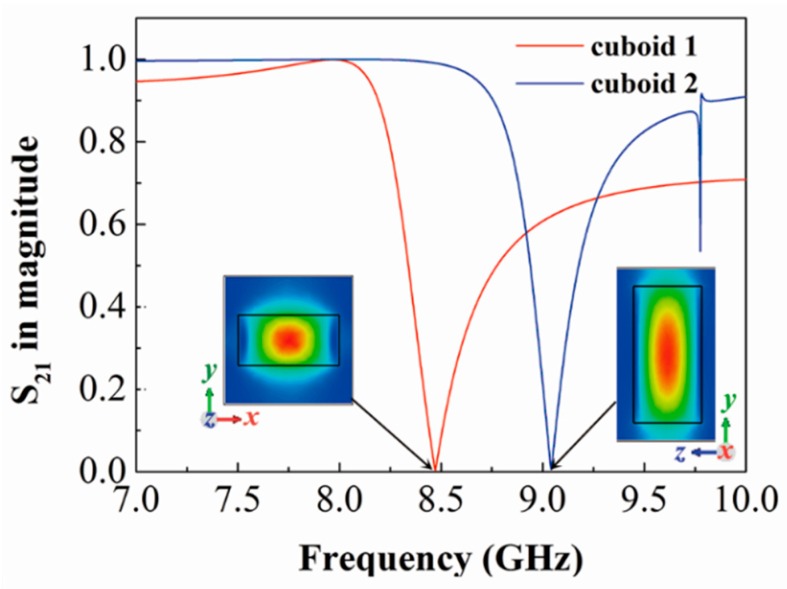
Simulated transmission spectra of the two single cuboids. The left inset is the schematic magnetic field |*H*| of cuboid 1 at 8.47 GHz, and the right inset is that of cuboid 2 at 9.04 GHz. The incident polarization is in the *x* direction.

The magnetic field distributions at 8.571 GHz for the *x*-polarized and the *y*-polarized waves are shown in Figure 3c,d. When the incident electric field is in the *x* direction, the magnetic field is highly concentrated in only one cuboid. This resonator behaves as a magnetic dipole, which is in the same direction as the incident magnetic field. Another cuboid is weak-resonant, but coupled to the strong dipole, which is a typical Fano interference. When the incident electric field is along the *y* direction, the two cuboids are strongly coupled, as illustrated in Figure 3d. Moreover, the transmission spectra (S_21_) of the two single cuboids are simulated when the incident electric field is in the *x* direction, and the schematic magnetic field distributions at the resonance peaks were also calculated, as depicted in Figure 4. The Mie resonance of the cuboids are at 8.47 GHz and 9.04 GHz. Both of them show magnetic dipole behaviors judging from the field distributions. The results coincidence with the 1st-order Mie resonance response given by the Mie scattering theory [39]. A small Fano resonance of the blue curve that emerged at 9.775 GHz, which is formed by two anti-parallel magnetic dipoles (see Supplementary Materials). It should be noted that the spectra of these two cuboids will exchange because of their symmetries, if the incident electric field is in the *y* direction. The Mie resonances of the two single cuboids exist in the vicinity of the Fano-type resonances mentioned above. Thus, we can derive that the Mie resonances can play an important role in the excitation of the Fano modes. The incident electromagnetic wave stimulates a strong localized field in one cuboid, and then excites the other cuboid through near-field coupling. Such a mechanism is established near the Mie resonances of these two cuboids with a strong coupling strength, which can be seen if we compare Figure 4 with Figure 3a. If one cuboid’s Mie resonance in the *x*-direction and another cuboid’s Mie resonance in the *y*-direction are of the same frequencies, the strongest AT effect will be acquired due to their mutual excitation. One outcome of this excitation process is that notable rotation of the polarization occurs in the proposed metamaterial with identical cuboids.

**Figure 5:**
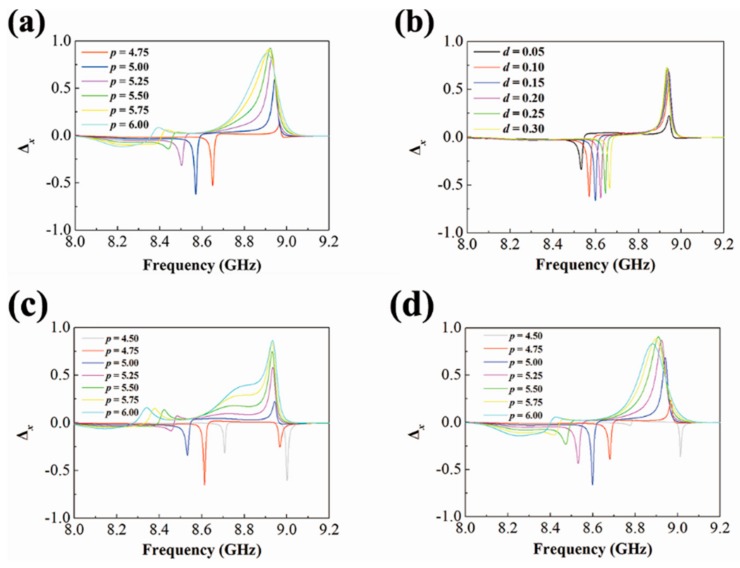
Dependence of the asymmetric parameter, Δ*_x_*, on the two geometrical parameters. (**a**) The lattice constant, *p*, when *d* = 0.10 mm. (**b**) The thickness of the spacer, *d*, when *p* = 5.0 mm. (**c**) The lattice constant, *p*, when *d* = 0.05 mm. (**d**) The lattice constant, *p*, when *d* = 0.15 mm.

Parameter sweeps were utilized to determine the dependence of the asymmetric parameter on the geometrical parameters. To study the influence of the lattice constant, the thickness of the dielectric spacer remains unchanged as *d* = 0.10 mm. In Figure 5a, the Δ*_x_* spectra vary when the lattice constant rises from 4.5 mm to 6.0 mm, suggesting that the interference between the metamolecules somehow cannot be ignored in this situation. The optimal value of *p* towards the AT effect is around 5 mm. As shown in Figure 5b, the thickness of the dielectric spacer also influences the AT response when *p* remains as 5.0 mm, because the interplay between the two cuboids depends on the gap. From the diagram, we can conclude that *d* = 0.15 mm is nearly the optimum. Besides, we also performed period sweeps for *d* = 0.05 mm and *d* = 0.15 mm. In Figure 5c, the asymmetric parameter reaches its maximum near *p* = 4.75 mm. When *d* = 0.15 mm, the largest value appears near *p* = 0.15 mm. The relation between Δ*_x_* and *p* varies for different *d* values. Though the function can be rather complicated, the simulation results suggest that the current configurations are the best design when we focus on the first Fano-peak.

Based on the Mie theory [39], for a lossless dielectric sphere, the frequency of 1st-order Mie resonance is given by:

(6) $$f=c_{0}\theta /2\pi R_{sp}\sqrt{\varepsilon_{sp}\mu_{sp}}$$

where *ε_sp_* and *μ_sp_* are the permittivity and the permeability of the sphere; *R_sp_* is the sphere’s radius; *c*_0_ is the velocity of light in vacuum; *θ* is nearly equal to *π* [40]. Similarly, the Mie resonance frequency is strongly related to the dielectric constant and the dimensions of the cuboid, which will provide more freedom for controlling and optimizing the AT response of the proposed metamolecule. For example, in using dielectrics with a high temperature coefficient of permittivity one can design a metamaterial with a thermal tunable AT effect. In addition, if the dimensions of the dielectric resonators are scaled down to submicrons to properly select materials [41], the implementation of our design at optical frequencies is possible.

## 4. Conclusions

In conclusion, we numerically proposed and demonstrated the AT phenomenon in a Mie-based dielectric metamaterials with Fano resonance. Two identical cuboids formed the chiral metamolecule, leading to a dual-band AT effect. Fano resonances were found near the AT transmission peaks. The simulated field distributions reveal that the metamolecule exhibits different inner coupling when the polarization of the applied wave is changed. The two cuboids give the 1st-order Mie resonances based on simulation and the AT effect emerges near these resonances. The parametric sweeps also suggest that controlling the interplay between the metamolecules or inside the metamolecule can optimize the electromagnetic response of the metamaterial. This study has potential for exploring high-performance polarization rotators in metamaterials.

## Supplementary Materials

The following are available online at https://www.mdpi.com/1996-1944/12/7/1003/s1, Figure S1: Electric field distribution at 9.775 GHz of the single cuboid; Figure S2: Magnetic field distribution at 9.775 GHz of the single cuboid.
