# Supplementary material for: Asymmetric Transmission in a Mie-Based Dielectric Metamaterial with Fano Resonance
Source: Materials (Basel). 2019 Mar 27;12(7):1003. doi: 10.3390/ma12071003 (PMC6480162; doi:10.3390/ma12071003)
Supplement: Supplementary file 1 [file materials-12-01003-s001.pdf]

materials

Article

Asymmetric Transmission in a Mie-Based Dielectric Metamaterial with Fano Resonance

Xiaobo Wang, Haohua Li and Ji Zhou *

State Key Laboratory of New Ceramics and Fine Processing, School of Materials Science and Engineering, Tsinghua University, Beijing 100084, China; wangxb14@mails.tsinghua.edu.cn (X.W.); lhh14@mails.tsinghua.edu.cn (H.L.)

* Correspondence: zhouji@mail.tsinghua.edu.cn; Tel.: +86-010-627-95384

Received: 5 March 2019; Accepted: 26 March 2019; Published: 27 March 2019

To investigate the Fano resonance in the blue curve of Figure 4. The field distributions at 9.775 GHz of the cuboid 2 have been simulated. From the following Figures S1 and S2, two anti-parallel magnetic dipoles are seen inside the cuboid, which is a typical destructive state.

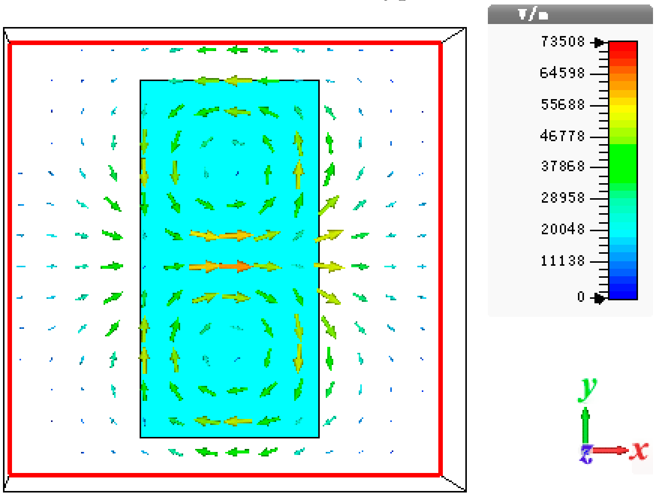

Figure 1. Electric field distribution at 9.775 GHz of the single cuboid.

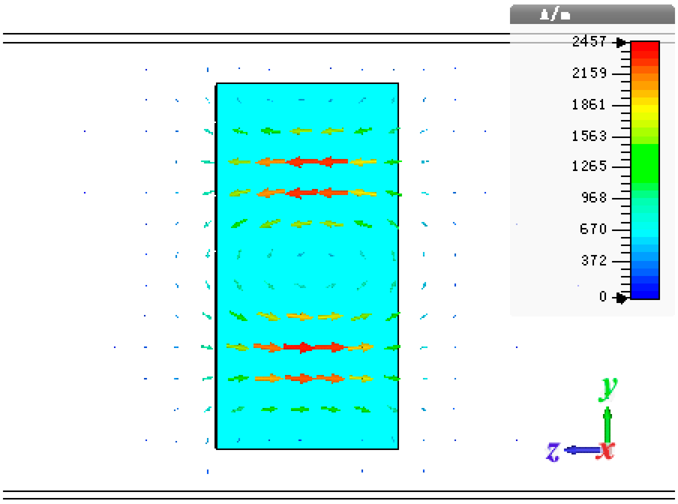

Figure 2. Magnetic field distribution at 9.775 GHz of the single cuboid.
